# The published trend of studies on COVID-19 and diabetes: bibliometric analysis

**DOI:** 10.3389/fendo.2023.1248676

**Published:** 2023-10-03

**Authors:** Yuanyuan Li, Lei Peng, Wei Gu

**Affiliations:** ^1^ Department of Endocrinology, Children’s Hospital of Nanjing Medical University, Nanjing, China; ^2^ Department of Gastroenterology, First Affiliated Hospital of Nanjing Medical University, Nanjing, China

**Keywords:** diabetes, COVID-19, bibliometric analysis, scientific collaboration, research trends

## Abstract

**Background:**

Since the COVID-19 pandemic outbreak, diabetes mellitus (DM) has been at the core of the confirmed risk factors for fatal or critical care unit-treated COVID-19 and COVID-19 related complications. Although relevant studies on DM have developed rapidly during the COVID-19 pandemic. However, the aforementioned research results have not been systematically quantified by means of bibliometric analysis.

**Purpose:**

The purpose of this study is to provide a comprehensive analysis of the current status and trends of publications related to DM research during the COVID19 epidemic.

**Methods:**

A bibliometric analysis was performed using the Web of Science database. In this study, we used citespace, R software and R-Bibliometrix to analyze keywords, most-cited authors, most-cited countries, most-cited global documents, and co-occurrence and co-citation networks.

**Results:**

A total of 1688 publications was included in this study. Investigators from the United States contributed the most publications. The United States, China and Europe have the most collaboration with the other countries/regions. A total of 3355 institutions made contributions to this study. Of the top 10 institutions with the most publications, N8 Research Partnership showed the most centrality. Among the top 10 journals, Diabetes Research and Clinical Practice published the most articles. Among authors included, Khunti Kamlesh is rated first with 27 papers and has the highest centrality. The most frequently co-cited article is entitled “Clinical course and risk factors for mortality of adult inpatients with COVID-19 in Wuhan, China: a retrospective cohort study”. The most popular keywords included diabetes, mortality, diabetes, outcome, occurrences, risk, and type 1 diabetes.

**Conclusion:**

This bibliometric study provides an overall picture of DM research and research trends during the COVID-19 pandemic and provides a basis for researchers to develop their next research strategies.

## Introduction

The SARS-CoV-2 virus is acknowledged to be the causative agent for the acute respiratory infectious disease which has been named as coronavirus disease 2019 (COVID-19) by The World Health Organization Since 2019 (1, 2). In the three years to 24 June 2023, over 640 million clinically diagnosed infections have been reported on a global scale with 6.6 million coronavirus deaths, according to World Health Organization survey (3). To individuals, infection of COVID-19 wreaks havoc on health, and it also contributes an enormous burden to national health delivery system. We have witnessed high incidence of susceptibility crowd of COVID-19. Nonetheless, the prognosis of the elderly and patients with chronic diseases, such as cardiovascular or respiratory dysfunction, diabetes and cancer, seems to be significantly worse comparing with others with COVID-19 infection (4, 5).

By conducting researches of different scales in people of different racial origins, diabetes, as one of the most common chronic diseases worldwide, has been proven to be one independent risk factor associated with critical infection of COVID-19. Due to the damage of immune response, diabetic patients are susceptible to diverse types of infection, and may be at heightened risk for severe illness and more death (6). In a large-scale observational study conducted by Sweden, the proportion of critical cases with T2D was reported to be more than that in non-T2D patients after adjusting for age, gender socio-demographic factors, drug treatment and multiple comorbidities (7). Although the pathophysiological mechanisms between COVID-19 and diabetes are still being further explored, studies have confirmed the existence of a bidirectional interaction between the two disease states, with the relevant pathways mainly involving stress-induced pathways.

Bibliometric analysis is an interdisciplinary approach to conduct quantitative literature research. Based on published literature and references, researchers use this statistical analysis tool to establish connections between published literature and research hotspots and trends in certain academic fields, thus providing a quantitative investigation of the trends of a research topic (8). Compared with traditional reviews, bibliometric analysis has shown greater advantages in objectively presenting the internal conceptual structure and potential associations of a large body of literature. Despite the extensive research on DM conducted by scholars during the COVID-19 pandemic, there is still a lack of quantitative analysis to show the current status of the DM research literature related to COVID-19 to have a complete understanding of the relationship between the two. We aim to predict the publication trends in this research area by analyzing the countries, institutions, partnerships, co-cited papers and keywords of the published DM-related articles during the COVID pandemic based on the current research results.

## Method

### Data sources and search strategies

**Figure 1:**
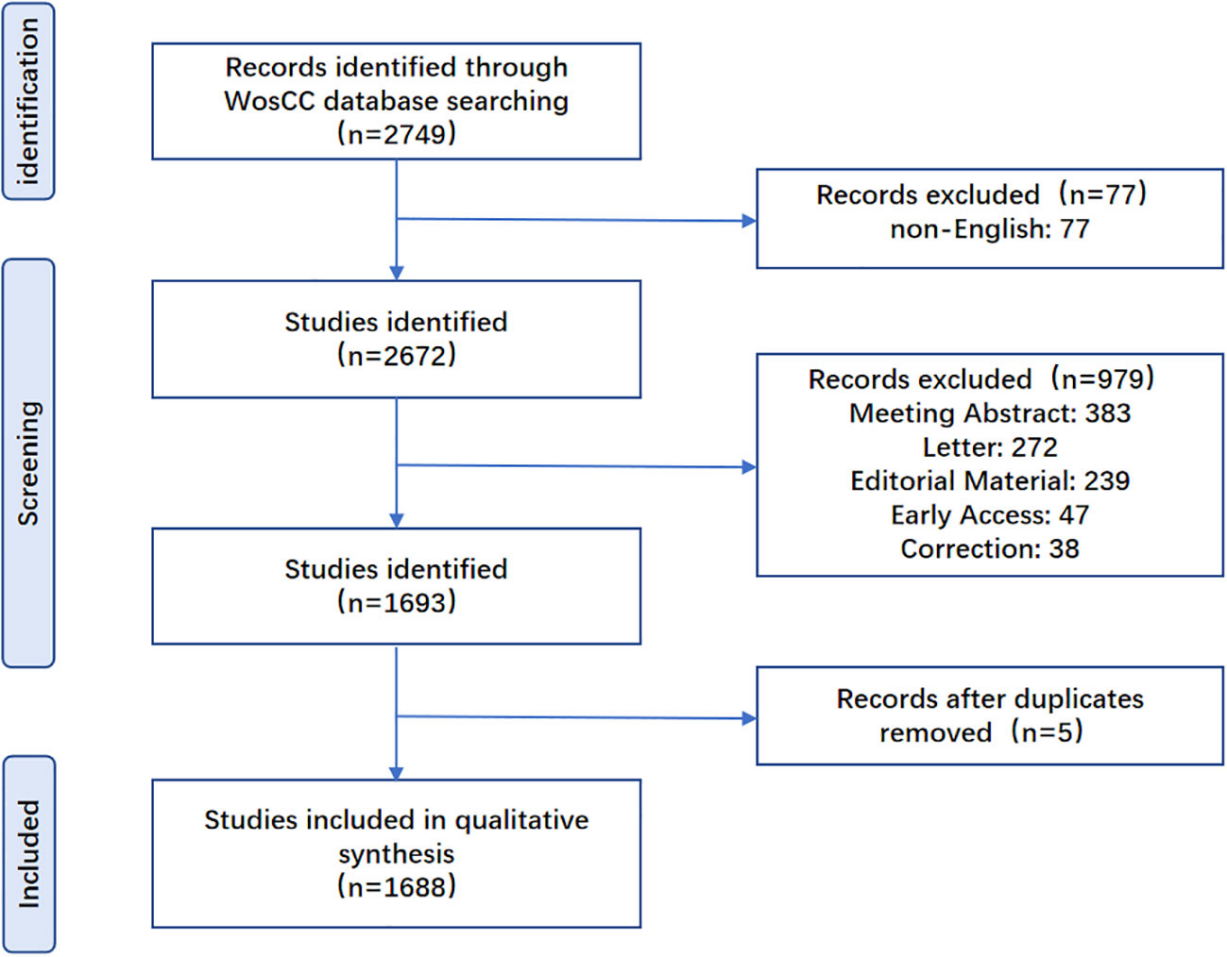
Flow chart of the data identification and screening results.

Web of Science Core Collection (WOS) database is commonly adopted to be used in bibliometric analysis, which provides comprehensive and multidisciplinary information statistically analysis (9). We comprehensively searched the included literature in the WOS database by publication time. All the relevant articles related to Diabetes and COVID-19 from 2019 to 2023 were searched (on June 24, 2023) by use of MeSH words. In this study, the following retrieval strategy was used: TI=(diabetes) AND (TI=(COVID 19) OR TI=(2019 novel coronavirus) OR TI=(coronavirus19) OR TI=(coronavirus disease 2019) OR TI=(2019-novel CoV) OR TI=(2019 ncov) OR TI=(COVID 2019) OR TI=(coronavirus 2019) OR TI=(nCoV-2019) OR TI=(ncovid19) OR TI=(2019-ncov) OR TI=(COVID-19) OR TI=(Severe acute respiratory syndrome coronavirus 2) OR TI=(SARS-CoV-2)).Literature restricted to the language and article type were further excluded. The detailed exclusion criteria were listed as follows: (1) meeting abstract, letter, editorial material, early access, corrections that were published as articles (2) the article was not written in English. Along the line, two researchers performed the literature search separately. **Figure 1** showed the research flow chart.

### Statistics and analysis

CiteSpace (6.1.R3) was used to analyze the included literature with the strongest citation bursts. By using CiteSpace (6.1.R3), co-citation analysis on countries, regions or institutions, co-citation analysis, biplot overlay of journals, and timeline view were performed. VOSviewer (1.6.18) was employed to provide visualization of publicly available data via analysis of bibliographic coupling, co-referencing or co-authorship affiliations. We carried out trend plots by analyzing the keywords’ frequency using R software and the Bibliometrix package.The Bibliometrix package also was used to show the evolution of keyword topics over time and to make visualization of the features of the published issues.

## Results

### General characteristics of publications

As shown in **Figure 1**, a review of papers published from 2019 to 2023 was conducted, and 2749 publications were available by the search terms. Then we screened out 77 publications in languages other than English. Simultaneously, 979 publications (including conference abstracts, letters, editorial materials, early access, and corrections) were filtered out, resulting in a total of 1693 publications afterwards. After loading the data into CiteSpace, five publications in which there were formatting errors or duplicates were filtered out, resulting in a total of 1688 publications for inclusion in this study. The included publications had a total of 28921 citations, with an average of 17.13 citations per paper, and an H-index of 73.

### Countries/regions

**Figure 2:**
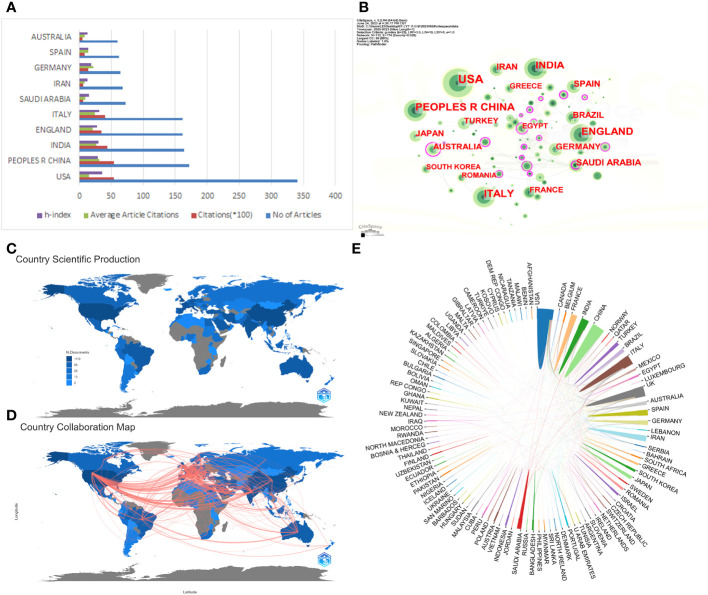
Cooperation network between countries. **(A)** The total publication number, total citations, average citation per paper, and H-index of the 10 most productive countries/regions; **(B)** The country collaboration network generated by Citespace; **(C, D)**: The country collaboration plotted on the world map; **(E)** Collaborative research between countries.

Publications included were from 112 countries or regions. Investigators from the United States contributed the most publications (n=341, 20.20% of the total; 5387 citations, mean 15.80 citations per paper), followed by China (n=172, 10.19%; 5423 citations, mean 31.53 citations per paper) and India (n=164, 9.72%; 4402 citations, mean 26.84 citations per paper) (**Figure 2A**). A total of 354 links and 112 nodes are depicted in **Figure 2B** to show the collaborative network between countries/regions. Each node symbolizes a country/region with a size proportional to the number of publications. The links between nodes represent the extent of collaboration between countries/regions. Of the top 10 countries/regions publishing the most papers, the Australia(0.24) displayed with the highest centrality, with USA and Italy(0.06) being the next highest, as shown in **Table 1**. **Figures 2C, D** display international collaboration between nations/regions. Of the top 10 published countries/regions, China, the United States and European countries have the most collaboration with the other countries/regions. **Figure 2E** shows that the tendency for global collaboration has become evident in targeting COVID-19.

**Table 1:** Top 10 countries/regions publishing the most papers in the field.

Rank	Country	No of Articles	Citations	Average Article Citations	H-index	Frequence	MCP_Ratio	Centrality
1	USA	341	5387	15.80	36	20.20%	0.177	0.06
2	PEOPLES R CHINA	172	5423	31.53	29	10.19%	0.151	0
3	INDIA	164	4402	26.84	30	9.72%	0.159	0
4	ENGLAND	162	3435	21.20	28	9.60%	0.355	0.03
5	ITALY	162	4015	24.78	31	9.60%	0.183	0.06
6	SAUDI ARABIA	72	654	9.08	15	4.27%	0.346	0.03
7	IRAN	68	559	8.22	13	4.03%	0.23	0
8	GERMANY	64	1450	22.66	19	3.79%	0.268	0
9	SPAIN	62	874	14.10	14	3.67%	0.114	0.03
10	AUSTRALIA	60	505	8.42	13	3.55%	0.257	0.24

### Institutions

**Figure 3:**
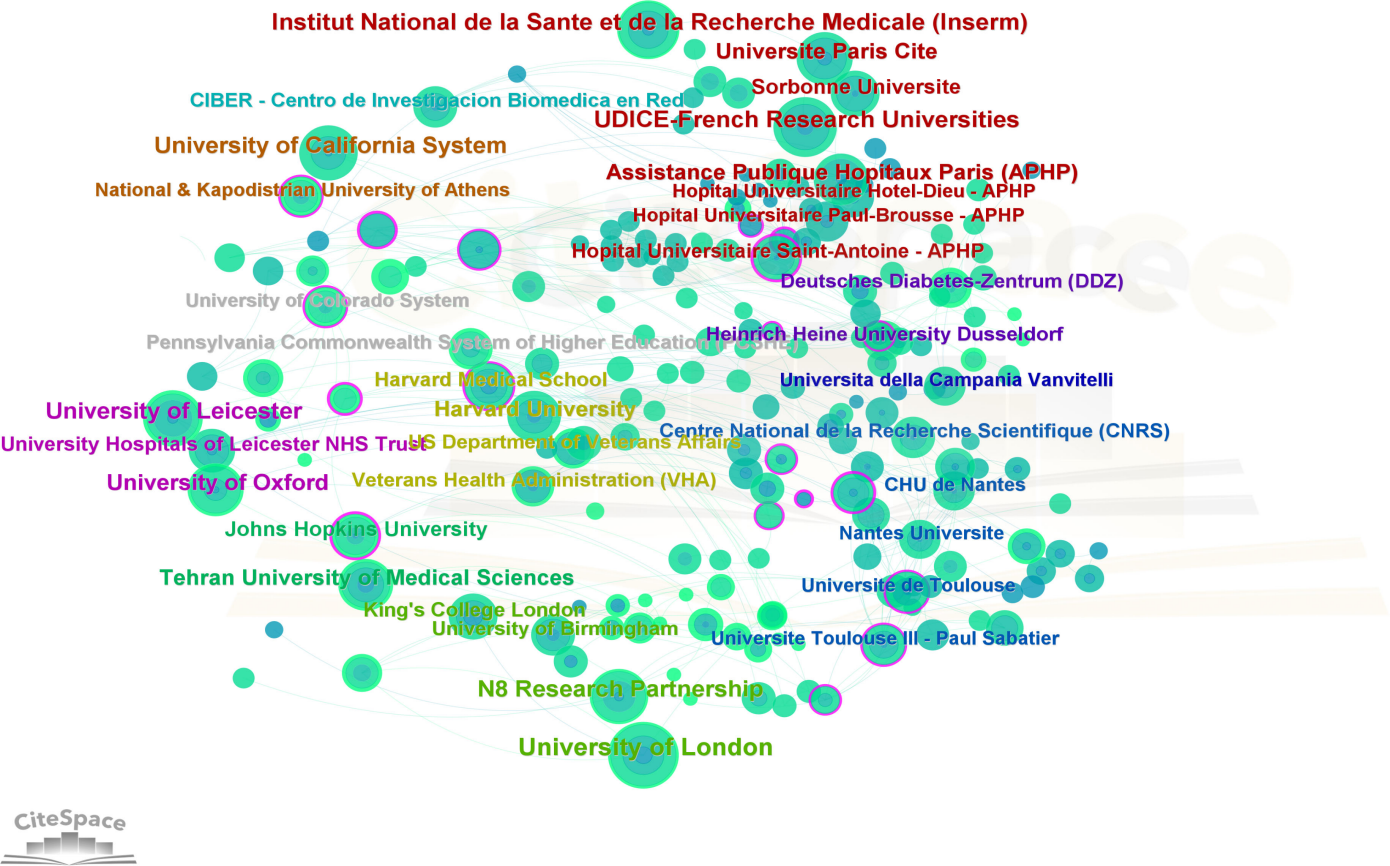
Cooperation network between major institutions.

A total of 3355 institutions worldwide made contributions to these 1688 publications. CiteSpace generated a graphical visualization of the network of institutional collaborations, as shown in **Figure 3**. Those top 5 institutions with the most papers (**Table 2**) were the University of London, UDICE French Research University, Egyptian Knowledge Bank (EKB), Huazhong University of Science Technology, Institut National De La Sante Et De La Recherche Medicale (INSERM). The betweenness centrality (BC) value is a metric to assess the nodes’ importance in a collaborative network. Of the top 10 institutions with the most publications, N8 Research Partnership showed the highest centrality (0.03), which was followed by the University of London, Universite Paris Cite and University of Oxford (0.02) as the most collaboration-oriented university.

**Table 2:** Top 10 institutions with the most papers in the field.

Rank	Institution	No of Articles	Citations	Average Article Citations	H-index	Frequence	Centrality
1	UNIVERSITY OF LONDON	44	651	14.80	14.8	2.61%	0.02
2	UDICE FRENCH RESEARCH UNIVERSITIES	37	1431	38.68	17	2.19%	0
3	EGYPTIAN KNOWLEDGE BANK EKB	35	426	12.17	12	2.07%	0
4	HUAZHONG UNIVERSITY OF SCIENCE TECHNOLOGY	34	3188	93.76	19	2.01%	0
5	INSTITUT NATIONAL DE LA SANTE ET DE LA RECHERCHE MEDICALE INSERM	34	1200	35.29	14	2.01%	0
6	UNIVERSITY OF LEICESTER	33	1579	47.85	14	1.96%	0
7	UNIVERSITY OF CALIFORNIA SYSTEM	32	549	17.16	11	1.90%	0.01
8	N8 RESEARCH PARTNERSHIP	31	299	9.65	11	1.84%	0.03
9	UNIVERSITE PARIS CITE	28	1305	46.61	15	1.66%	0.02
10	UNIVERSITY OF OXFORD	28	379	13.54	10	1.66%	0.02

### Authors and co-citation authors

**Figure 4:**
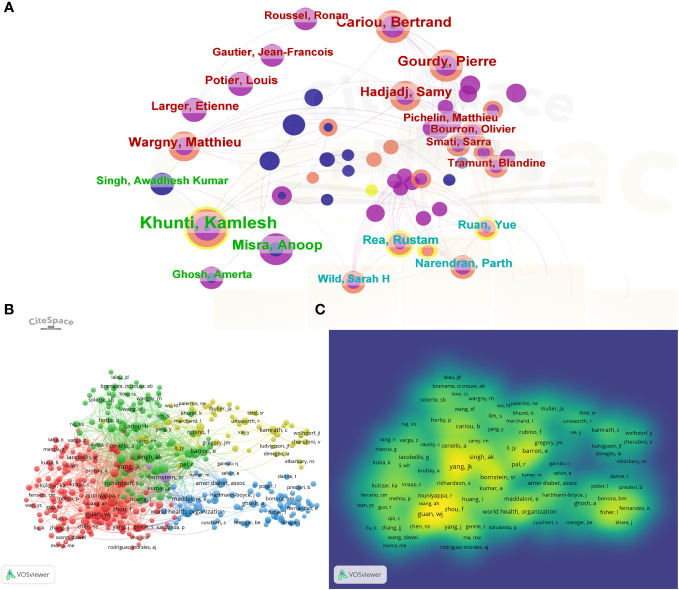
Map of collaboration networks of co-author analysis **(A)** Network visualization map of authors; **(B, C)** Overlay visualization map of authors. Network map showing authors’ collaborations.

**Figure 4A** delineates the collaborative network among authors who authored more than 3 papers, with 334 authors conducting research on diabetes and COVID-19 from 2019 to 2023. In **Table 3**, Khunti Kamlesh is rated first with 27 papers. He has the most published papers in the field. and has the highest centrality (0.04). Through analysis of authors’ co-citation networks, those with more than 20 citations were defined as key researchers (**Figures 4B, C**): Connections indicate collaboration between authors, and the size of the circles represents the amount of citations. Total link strength (TLS) indicates the impact of an author’s publication on other contributing authors. The highest number of co-citations was recorded for Yang JK (n=410), followed by Guan WJ (n=327). The top 3 authors having the highest TLS were Yang JK, Guan WJ and Zhou F (**Table 3**).

**Table 3:** Leading authors in the field.

Rank	Author	No of Articles	Citations	Average Article Citations	H-index	Frequence	Centrality
1	Khunti K	27	1540	57.04	12	1.60%	0.04
2	Cariou B	16	855	53.44	9	0.95%	0.01
3	Misra A	16	1554	97.13	12	0.95%	0.01
4	Gourdy P	15	854	56.93	9	0.89%	0.01
5	Wargny M	15	854	56.93	9	0.89%	0.01
6	Hadjadj S	14	845	60.36	8	0.83%	0.01
7	Holl RW	13	103	7.92	6	0.77%	0
8	Schiaffini R	12	288	24.00	7	0.71%	0
9	Schaan BD	11	158	14.36	4	0.65%	0
10	Yang Y	11	410	37.27	5	0.65%	0
Co-citation authors in the field							

Rank	Author	Co-citations	Total link strength				
1	Yang Jk	410	10181				
2	Guan Wj	327	6915				
3	Zhou F	303	6557				
4	Singh Ak	295	6522				
5	Guo Wn	250	5613				
6	Bornstein Sr	244	5613				
7	Pal R	243	4975				
8	Fadini Gp	188	4858				
9	Sardu C	181	4725				
10	Cariou B	202	4664				

### Journals

**Figure 5:**
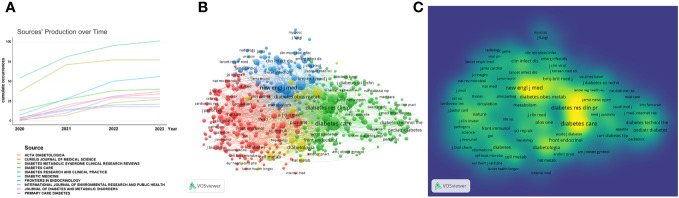
Map of collaboration networks of journal analysis. **(A)** The annual and cumulative numbers of research articles of journals; **(B, C)** The network visualization diagram of journal co-citation analysis of generated by VOSviewer. Overlay visualization map of journals.

From 2019 to 2023, 1,688 research articles related to COVID-19 and diabetes were published in 512 journals, 74 of which contained at least 5 articles. The 10 journals with the most published articles are listed in **Table 4**. Diabetes Research and Clinical Practice published the most articles (n=101), followed by Diabetes Metabolic Syndrome Clinical Research and Frontiers in Endocrinology (n=77 and 56, respectively). Additionally, the annual incidence of these 10 journals was generated by R-Bibliometrix to get a more specific picture of the trends in the number of publications of these journals across years (**Figure 5A**). A network visualization of the journal co-citation analysis was produced by VOS viewer, as shown in **Figures 5B, C**. Only journals that were visually cited at least 20 times were listed. Among the 461 journals that met the criteria, the top 3 journals that were frequently co-citation were DIABETES CARE (3435 times), DIABETES RESEARCH AND CLINICAL PRACTICE (1980 times), and NEW ENGLAND JOURNAL OF MEDICINE (1573 times) (**Table 4**).

**Table 4:** Top 10 journals with the most published and co-cited articles in the field.

Rank	Journal	No of Articles	Citations	Average Article Citations	h-index	Frequence
1	*DIABETES RESEARCH AND CLINICAL PRACTICE*	101	2399	23.75	27	5.98%
2	*DIABETES METABOLIC SYNDROME CLINICAL RESEARCH REVIEWS*	77	3858	50.10	27	4.56%
3	*FRONTIERS IN ENDOCRINOLOGY*	56	641	11.45	15	3.32%
4	*DIABETES CARE*	40	2095	52.38	23	2.37%
5	*PRIMARY CARE DIABETES*	37	309	8.35	10	2.19%
6	*ACTA DIABETOLOGICA*	34	526	15.47	14	2.01%
7	*INTERNATIONAL JOURNAL OF ENVIRONMENTAL RESEARCH AND PUBLIC HEALTH*	29	108	3.72	6	1.72%
8	*CUREUS JOURNAL OF MEDICAL SCIENCE*	27	33	1.22	4	1.60%
9	*DIABETIC MEDICINE*	21	339	16.14	10	1.24%
10	*JOURNAL OF DIABETES AND METABOLIC DISORDERS*	18	104	5.78	6	1.07%
Co-citation journals in the field						

Rank	Journal	Co-citations	Total link strength			
1	*DIABETES CARE*	3435	143531			
2	*DIABETES RES CLIN PR*	1980	85886			
3	*NEW ENGL J MED*	1573	76800			
4	*LANCET*	1461	70439			
5	*DIABETES METAB SYND*	1789	68390			
6	*JAMA-J AM MED ASSOC*	1308	62039			
7	*LANCET DIABETES ENDO*	1250	54733			
8	*DIABETOLOGIA*	908	47521			
9	*PLOS ONE*	672	37856			
10	*NATURE*	534	36196			

### Dual-map overlays

**Figure 6:**
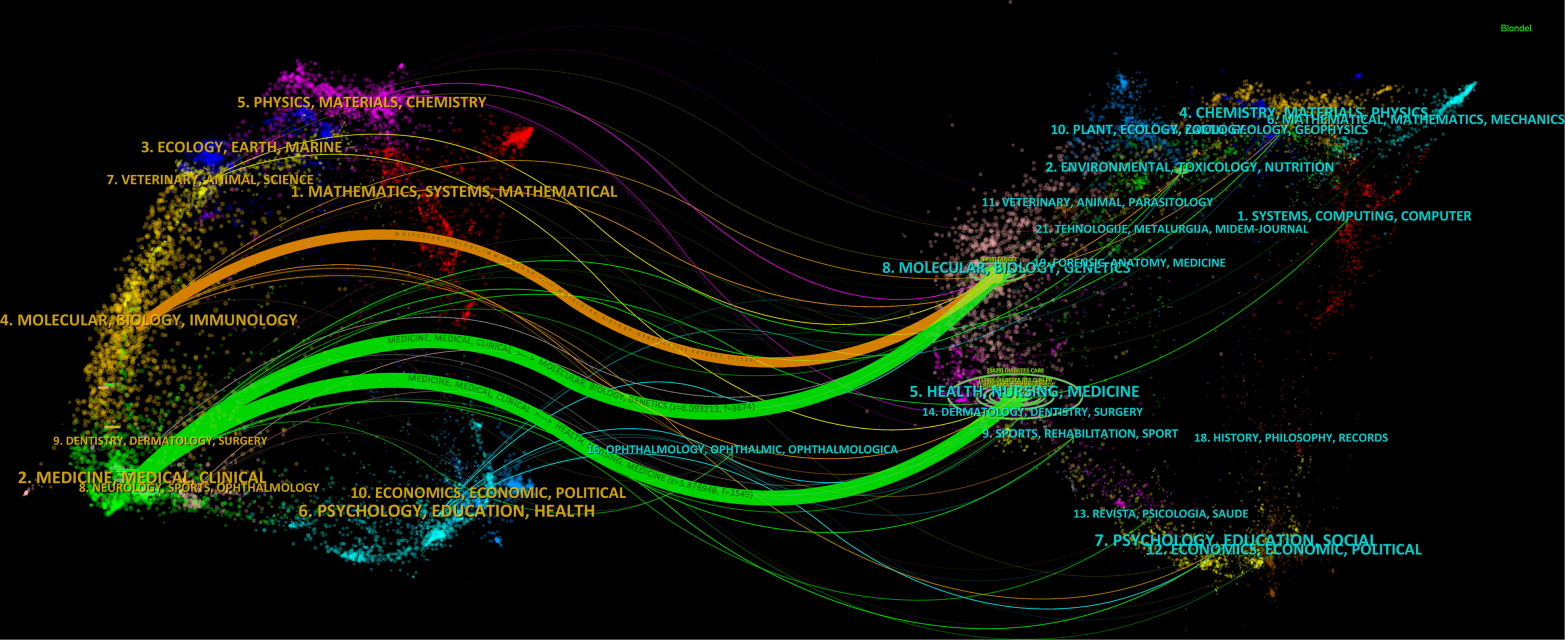
Dual map overlay of journals that contributed to publications on the use of artificial intelligence.

The literature of cited journals makes up the reference knowledge base, and the field of study of a highly cited journal represents an active interest or emerging field. We mapped and outlined the literature co-citation relationships for journal research areas using CiteSpace, with a graph of citing journals on the left and a graph of cited journals on the right. Remarkably, the colored paths shown in **Figure 6** represent citation relationships in fields of highly active research. Published articles are concentrated in journals in the area of medicine, medical, and clinical, while most of the cited articles are published in journals in the area of molecular, biology, genetic, and health, nursing, and medicine.

### Analysis of co-cited reference

**Figure 7:**
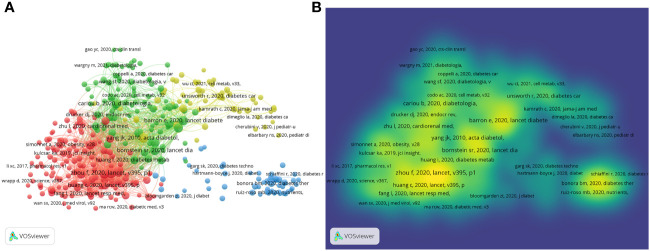
Map of co-cited references. **(A)** The network visualization diagram of references co-citation analysis; **(B)** References co-citation density chart.

In **Figures 7A, B**, a map of the 340 co-cited references that were cited more than 20 times is depicted. A cited reference is each represented by a node. The amount of co-cited references is characterized by the size of the node. Cross-references are indicated by the links between the nodes. The wider the connection, the higher the frequency of co-citations is indicated. The five most frequently co-cited references are listed in **Table 5**. The most frequently co-cited article is the article by Fei Zhou (2020) published in The Lancet (10), entitled “Clinical course and risk factors for mortality of adult inpatients with COVID-19 in Wuhan, China: a retrospective cohort study”. Then followed by Guo WN(2020), entitled “Diabetes is a risk factor for the progression and prognosis of COVID-19” was published in Diabetes Metab Res Rev (11); Yang JK(2010), entitled “Binding of SARS coronavirus to its receptor damages islets and causes acute diabetes” was published in acta diabetol (12); Barron E(2020), entitled “Associations of type 1 and type 2 diabetes with COVID-19-related mortality in England: a whole-population study” was published in lancet diabetes endo and Wu ZY (2020), entitled “Characteristics of and Important Lessons From the Coronavirus Disease 2019 (COVID-19) Outbreak in China: Summary of a Report of 72 314 Cases From the Chinese Center for Disease Control and Prevention” was published in JAMA (5, 13).

**Table 5:** Top 10 most frequently co-cited references.

Rank	Author	Year	Journal	Citations	Total link strength	Cited reference
1	Zhou F	2020	*LANCET*	297	4968	Clinical course and risk factors for mortality of adult inpatients with COVID-19 in Wuhan, China: a retrospective cohort study
2	Guo Wn	2020	*DIABETES-METAB RES*	250	4430	Diabetes is a risk factor for the progression and prognosis of COVID-19
3	Yang Jk	2010	*ACTA DIABETOL*	204	4008	Binding of SARS coronavirus to its receptor damages islets and causes acute diabetes
4	Barron E	2020	*LANCET DIABETES ENDO*	203	3204	Associations of type 1 and type 2 diabetes with COVID-19-related mortality in England: a whole-population study
5	Wu Zy	2020	*JAMA-J AM MED ASSOC*	201	3601	Characteristics of and Important Lessons From the Coronavirus Disease 2019 (COVID-19) Outbreak in China: Summary of a Report of 72 314 Cases From the Chinese Center for Disease Control and Prevention
6	Bornstein Sr	2020	*LANCET DIABETES ENDO*	198	3438	Practical recommendations for the management of diabetes in patients with COVID-19
7	Yang Jk	2006	*DIABETIC MED*	197	4026	Plasma glucose levels and diabetes are independent predictors for mortality and morbidity in patients with SARS
8	Zhu Lh	2020	*CELL METAB*	196	3262	Association of Blood Glucose Control and Outcomes in Patients with COVID-19 and Pre-existing Type 2 Diabetes

### Keywords

**Figure 8:**
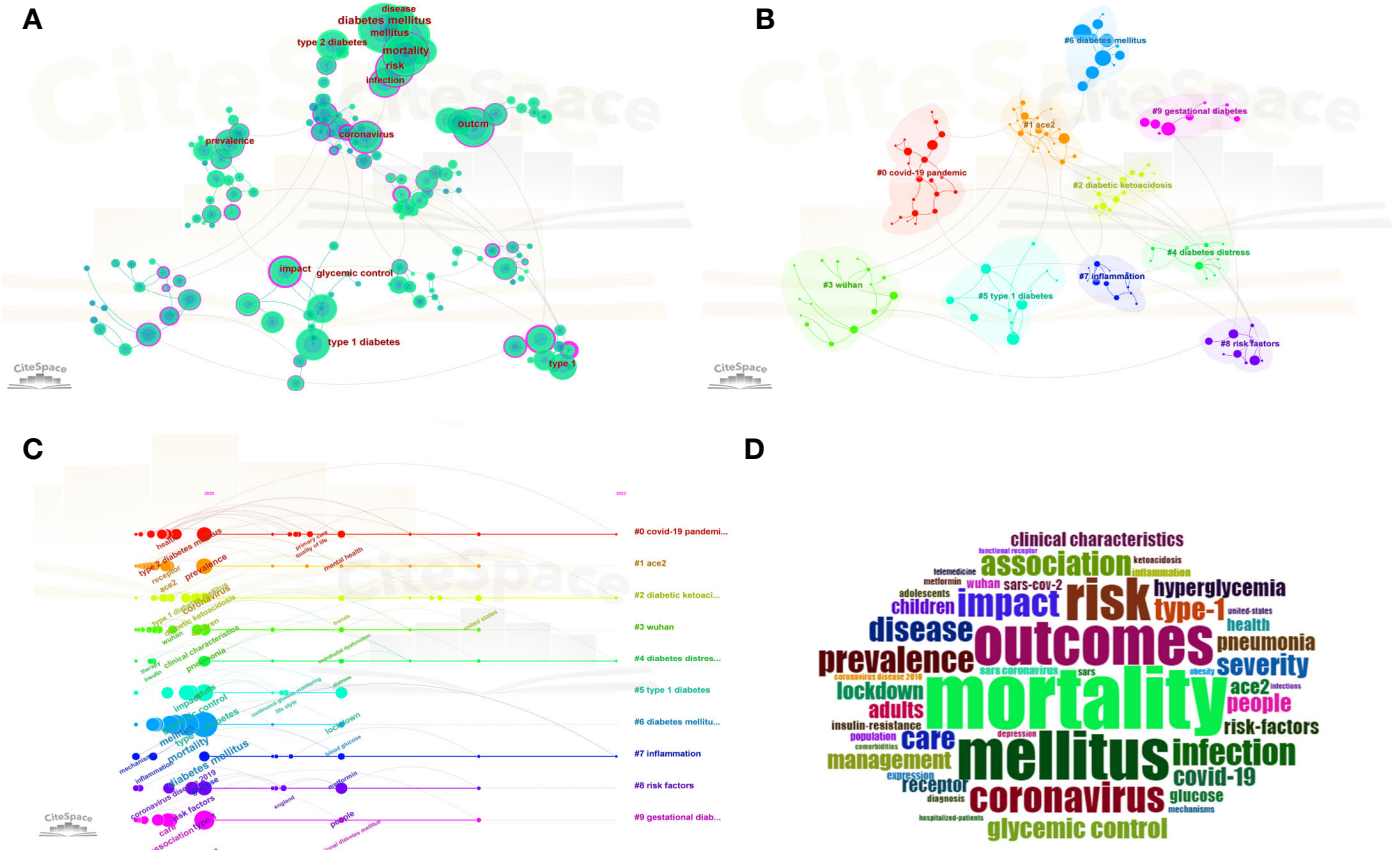
Map of Keyword clusters analysis. **(A)** Visualization of keyword co-occurrence; **(B)** map of Keyword clusters analysis; **(C)** timeline map of keyword; **(D)** word Cloud map regarding the keywords’ frequency of occurrence generated from R studio Documents menu of Biblioshiny package.

**Table 6** shows the frequency of common occurrences of the top 10 key words. The most frequent word was diabetes mellitus (330 occurrences), followed by mortality (249 occurrences), mellitus (195 occurrences), outcome (183 occurrences), risk (168 occurrences), and type 1 diabetes (144 occurrences). Keywords with high centrality indicate hot spots in the field, with values between 0 and 1. In terms of centrality, the top 10 keywords were England, risk factors, coronavirus, pneumonia, mortality, coronavirus disease 2019, onset, impact, mellitus and receptor. The keyword cluster plots for COVID-19 and diabetes are shown in **Figure 8A**. The analysis of keywords was based on log-likelihood test cluster analysis of keyword co-occurrence analysis. There were 10 clusters obtained in this study. Details are shown in **Figure 8B**. the Q value (cluster module value) was 0.7892, indicating a significant clustering structure. In addition, the S-value, the mean profile value, was 0.9418, which indicated that the cluster members were highly homogeneous. The first 10 keyword clusters were chosen for analysis and are presented in **Table 7**. they are “covid-19 pandemic”, “angiotensin-converting enzyme 2”, “diabetic ketoacidosis”, “ wuhan”, “diabetes distress”, “type 1 diabetes”, “diabetes mellitus”, “ inflammation”, “ risk factors”, “gestational diabetes”. The values for each cluster profile are greater than 0.5, suggesting a high degree of homogeneity and consistency in the clusters. **Figure 8C** shows the top 50 terms with the most intense outbreaks in the field. **Figure 8D** describes the trend of hot topics of the literature on COVID-19 and diabetes during 2020-2023.

**Table 6:** Frequency and centrality of the top 10 key words.

Ranked by frequency				
Rank	Frequency	Centrality	Year	Key word
1	330	0	2020	diabetes mellitus
2	249	0.2	2020	mortality
3	195	0	2020	mellitus
4	183	0.22	2020	outcome
5	168	0.22	2020	risk
6	144	0.07	2020	type 1 diabetes
7	131	0	2020	type 2 diabetes
8	124	0.36	2020	coronavirus
9	118	0.07	2020	glycemic control
10	110	0.45	2020	impact

Ranked by centrality				
Rank	Frequency	Centrality	Year	Key word
1	3	0.92	2023	england
2	86	0.76	2020	risk factors
3	124	0.72	2020	coronavirus
4	66	0.69	2020	pneumonia
5	249	0.68	2020	mortality
6	57	0.6	2020	coronavirus disease 2019
7	4	0.59	2023	onset
8	110	0.57	2020	impact
9	195	0.55	2020	mellitus
10	56	0.55	2020	receptor

**Table 7:** Top 10 keyword clusters in the field.

Cluster ID	Size	Silhouette	Cluster name
0	21	0.94	covid-19 pandemic
1	21	0.919	ace2
2	16	0.985	diabetic ketoacidosis
3	16	0.856	wuhan
4	12	0.98	diabetes distress
5	12	0.963	type 1 diabetes
6	11	1	diabetes mellitus
7	11	0.892	inflammation
8	10	0.894	risk factors
9	10	1	gestational diabetes

### Burstiness of keywords

**Figure 9:**
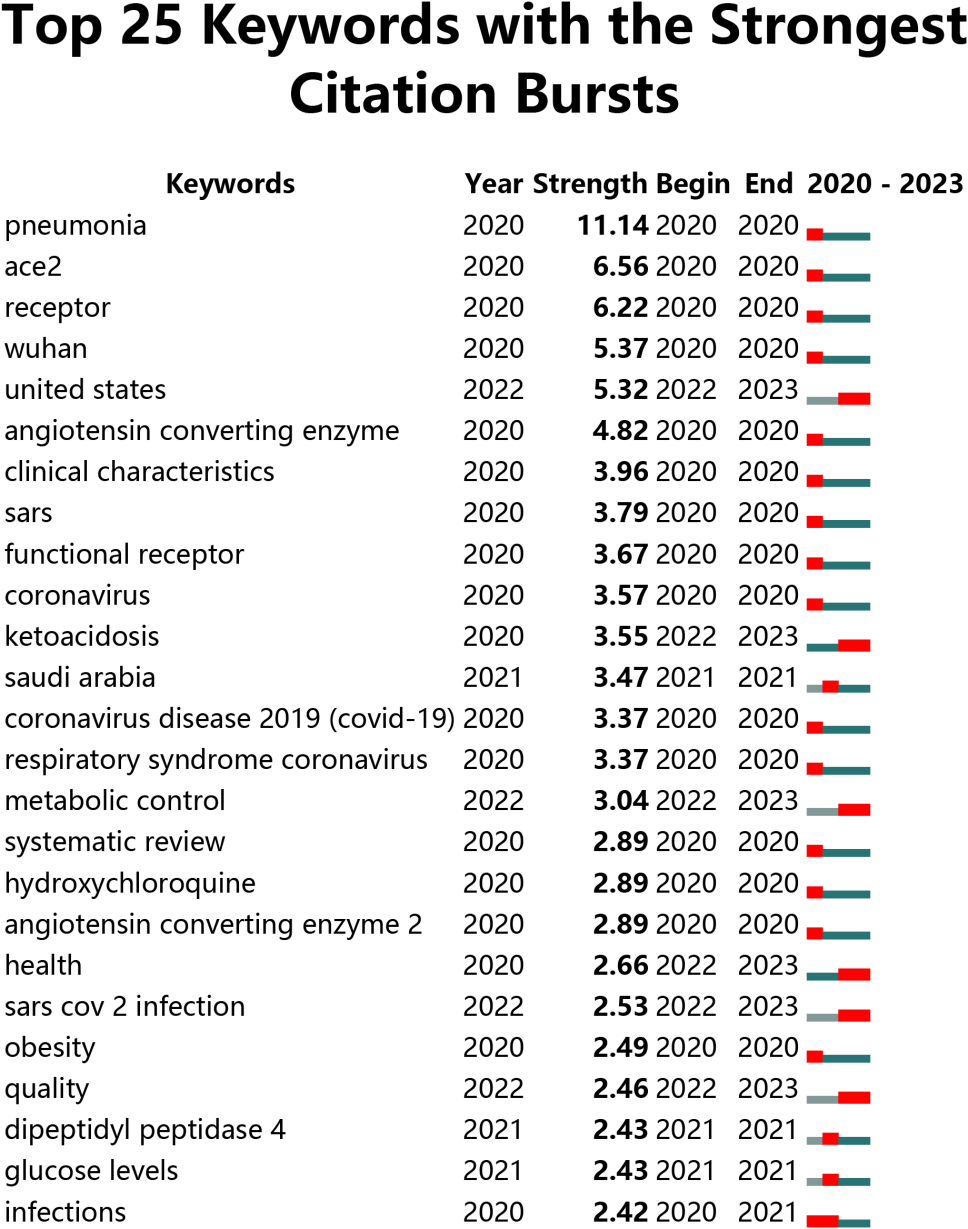
Visualization of Keyword Bursts.

CiteSpace was used for keyword burst detection (**Figure 9**). Keyword burstiness allows for representing new academic trends, foreshadowing future frontier research avenues, and highlighting potential topicalities in a discipline. Burstiness detection is shown as the red section of the blue timeline, indicating the onset year, finish year, and duration of the burst. A blue line is shown for the time line. We were particularly attracted to terms that were of research relevance in the top 25 keywords with the greatest outbreak intensity. These terms are representative of research trends for both the COVID-19 and diabetes fields (**Figure 8**). From 2020 to 2023, the highest outbreak intensity was observed for pneumonia (11), followed by receptors (6.56) and coronavirus (6.22). It is noteworthy that the burst of resistance and outbreak still continues.

## Discussion

Since the COVID-19 pandemic, diabetes has been identified as a significant risk factor for increased mortality from severe COVID. To investigate the relationship between these two disease states, researchers have conducted a large number of studies and published numerous articles. This is the first time that a bibliometric review of all publications related to DM and COVID-19 has been conducted. The most published journals and the most cited articles were identified. The collaboration relationships across countries are depicted, and significant subjects in the field of research are discussed. Analysis of the leading journals and the most cited articles worldwide assisted in identifying potentially influential articles in the area. The networks of collaboration, trending keywords and thematic trends can be used as a reference for future research. In this study, a collection of 1688 articles was collected from the Web of Science Core Collection database. The United States published 341 papers related to diabetes and COVID-19 in the past three years, making it the country with the most research in related fields, followed by China (172 papers) and India (164 papers). Although the US was higher than China in terms of total number of papers, China ranked higher than the US in terms of total and average citation rates. We note that the 172 papers published by Chinese researchers in high impact factor journals include a large number of highly cited articles, and two of them have citation rates above 500 (14), suggesting the strong academic influence of China in the relevant research areas.

According to the latest data from the International Diabetes Federation, there are about 463 million people with diabetes worldwide, and the number is expected to reach 550 million in 2030, with China ranking first in the world in the number of people with diabetes (15, 16). The prevention and treatment of diabetes has become one of the common public health problems faced by the world (16, 17) By analyzing the data collected by the World Health Organization (WHO), we found that consistent with the results of the bibliometric analysis of those other COVID-19 studies not related to DM, the United States had the most studies on DM and COVID-related aspects, followed by China. Through bibliometric statistical analysis, we found that the majority of articles in relevant research publications on DM and COVID were also from the United States and China (18, 19). The analysis of research collaboration networks helps researchers to scientifically assess the next step in the scientific collaboration process and to select key collaborators. The analysis of this study found that among countries, China, the United States, and Europe collaborate most closely with other countries/regions, which may be related to their better economic status and higher research expenditures. In contrast, other developing countries remain the weak link in the current global collaboration due to their poorer economic level and insufficient research investment and infrastructure. This information may be crucial for researchers to select countries for further study exchanges or research collaborations.

At the research institution level, University of London, UDICE French Research University, Egyptian Knowledge Bank (EKB), Huazhong University of Science Technology, Institut National De La Sante Et De La Recherche Medicale(INSERM) are the five institutions with the highest number of COVID-related publications among all institutions. In England, there is a close interaction and cooperation between institutions, and N8 Research Partnership shows a central position. In terms of the number of published articles, the top ten research institutions are mainly universities of various countries, which may be related to the fact that universities invest more time, energy and resources in scientific research than other institutions and their relative emphasis on talent development. As far as the authors are concerned, Khunti Kamlesh has published a total of 27 papers and has made significant contributions to the field related to COVID and DM. Khunti Kamlesh’s study on COVID-19 and DM primarily suggests that diabetes is an independent factor for in-hospital mortality in COVID-19 (5).

It was found that increased mortality associated with COVID-19 was associated with distant complications of diabetes, mainly with cardiovascular and renal complications. In addition, glycemic control and BMI were independent risk factors for elevated COVID-related mortality (20). Considering the continued epidemiological trend of COVID-19 and the continued increase in the prevalence of diabetic patients in the future, these findings are crucial for researchers to choose and adjust the future direction of their studies. By analyzing the co-cited articles of the authors, we found that Yang JK showed the greatest number of co-citations (n=410), followed by Guan WJ (n=327). Yang’s study demonstrated that diabetes and environmental hyperglycemia were independent predictors of morbidity and mortality in SARS patients. The prognosis of SARS patients can be improved by metabolic control (21). A study by Zhou published in The Lancet showed risk factors associated with in-hospital mortality in adults with COVID-19, which was confirmed by the laboratories of Jinyintan Hospital and Wuhan Pulmonary Hospital (10). The analysis of literature co-citation rates and frequency of keyword occurrences can help to understand the main research directions, research hotspots and their evolution in related fields (22).

Journal publication analysis can be useful in providing information to help researchers selecting appropriate journals for submitting their articles. In this research, the top 10 journals with 440 published articles related to DM and COVID-19 were found to be subspecialty journals. This could be due to the need for clinicians in this field to have subspecialty training. It is also exciting to note that subspecialty journals are not the only journals that publish relevant articles, but some general journals like Frontiers in Endocrinology also publish similar articles. Through in-depth analysis of keywords, we found that the primary keywords of high frequency in the present research focus on “COVID-19 and diabetes” were diabetes mellitus, mortality, mellitus, outcome, risk, and type 1 diabetes. The report shows that “increased COVID-19-related mortality associated with diabetes” is a hot topic for scholars these years. The high centrality of keywords such as “angiotensin-converting enzyme 2” and “inflammation” suggests that research on diabetes and COVID has partially shifted from epidemiology to pathogenesis studies. At this stage, there is also a significant increase in concern for adolescents and type 1 diabetes. Basic research and clinical trials related to the impact of the COVID-19 epidemic on adolescents and young adults (23), the effect on mood, leading to anxiety and depression (24), are actively being conducted along with the ongoing epidemic of COVID. Several investigations showed that non-enzymatic glycation of ACE2 receptors might be the pathogenic reason of the deteriorating outcome of COVID-19 disease in diabetic conditions (25, 26). Through analysis of keyword clusters, we identified that the hotspots of “COVID-19 and diabetes” research focus on type 1 diabetes and pathogenesis mechanism (angiotensin-converting enzyme 2, inflammation, cell). Recent studies have found increased morbidity and mortality in type 1 diabetes mellitus (T1DM) during the COVID-19 epidemic (27). The mechanism may be related to the excessive release of pro-inflammatory cytokines in the severe COVID-19 state. Notably, COVID-19 was demonstrated to lead to a severe imbalance in glucose homeostasis. Glucotoxicity can then synergize with inflammatory cytokine storms to promote oxidative stress, stimulate immune dysregulation, impair endothelial cell function and lead to a range of metabolic complications such as increased risk of thromboembolism and multi-organ damage, causing increased eventual patient mortality (28). It is a vicious circle. In addition, COVID-19 can bind to the angiotensin-converting enzyme 2 (ACE2) receptor in pancreatic β-cells thereby leading to pancreatic β-cell destruction, which in turn promotes the development of diabetes (29). Also, some natural products such as quercetin, curcumin or other hypoglycemic agents would participant in COVID-19 and diabetes research (30–32). Nevertheless, long-term follow-up studies are still needed to assess the impact of COVID on the incidence, type, and complications of diabetes.

## Conclusion

This article provides the first presentation of a bibliometric evaluation of the publications on diabetes and COVID-19. This study also has some limitations, mainly in database selection, literature omissions due to time point limitations and citation analysis bias due to self-referencing. Despite these limitations, this bibliometric study still provides an overall picture of DM research and research trends during the COVID-19 pandemic and provides a basis for researchers to develop their next research strategies.

## Data availability statement

The original contributions presented in the study are included in the article/supplementary material. Further inquiries can be directed to the corresponding authors.

## Author contributions

YL designed the study. YL and LP acquired the data and performed statistical analyses. YL and LP drafted the manuscript. YL and WG reviewed and edited the manuscript. All authors contributed to the article and approved the submitted version.
